# Retraction Note: Suppression of NF-κB signaling by ECN in an arthritic model of inflammation

**DOI:** 10.1186/s12906-023-03954-5

**Published:** 2023-04-20

**Authors:** Amna Khan, Li Zhang, Chang Hu Li, Ashraf Ullah Khan, Bushra Shal, Adnan Khan, Sajjad Ahmad, Fakhar ud Din, Zia ur rehman, Feng Wang, Salman Khan

**Affiliations:** 1 Pharmacological Sciences Research Lab, Department of Pharmacy, Faculty of Biological Sciences, Quaid-i-Azam University, Islamabad, Pakistan; 2 Department of Medical Oncology, Cancer Center, West China Hospital, West China Medical School, Sichuan University, Sichuan, People’s Republic of China; 3 Division of Radiation Physics, Cancer Center, West China Hospital, Sichuan University, Chengdu, 610041 Sichuan China; 4 Faculty of Pharmaceutical Sciences, Abasyn University, Peshawar, KPK Pakistan; 5 Department of Health and Biological Sciences, Abasyn University, Peshawar, 25000 Pakistan; 6 Department of Pharmacy, Faculty of Biological Sciences, Quad-i-Azam University, Islamabad, Pakistan; 7 Department of Chemistry, Quaid-I-Azam University, Islamabad, 45320 Pakistan

**Retraction Note: BMC Complement Med Ther 22, 158 (2022)**

**https://doi.org/10.1186/s12906-022-03629-7**

The Editor has retracted this article. After publication, concerns were raised regarding image similarities among the panels in Fig. 8. Specifically:

- The p-JNK ENC image appears to overlap with p-JNK Dexa;
- The NF-κB Normal control appears to overlap with NF-κB ECN.

Additionally, Fig. 8 TNF-a Dexa and ECN images appear to overlap with Fig. 13 TNF-a NCHDH and CFA in [1], respectively.

The authors have provided the raw data to address these concerns. However, further checks by the Publisher identified numerous irregularities in the raw data, and further cases of data overlap with [1]. The Editor therefore no longer has confidence in the presented data.

Salman Khan does not agree to this retraction. None of the other authors have responded to any correspondence from the editor or publisher about this retraction.
